# A New Semi-Analytical Solution for an Arbitrary Hardening Law and Its Application to Tube Hydroforming

**DOI:** 10.3390/ma15175888

**Published:** 2022-08-26

**Authors:** Stanislav Strashnov, Sergei Alexandrov, Lihui Lang

**Affiliations:** 1 General Education Courses Department, Peoples’ Friendship University of Russia (RUDN University), 6 Miklukho-Maklaya St, 117198 Moscow, Russia; 2 School of Mechanical Engineering and Automation, Beihang University, No. 37 Xueyuan Road, Beijing 100191, China; 3 Department of Civil Engineering, Peoples’ Friendship University of Russia (RUDN University), 6 Miklukho-Maklaya St, 117198 Moscow, Russia

**Keywords:** strain hardening, large strain, semi-analytical solution, tube hydroforming

## Abstract

The present study consists of two parts. The first part supplies an exact semi-analytical solution for a general model of rigid plastic strain hardening material at large strains. The second part applies this solution to tube hydroforming design. The solution provides stress and velocity fields in a hollow cylinder subject to simultaneous expansion and elongation/contraction. No restriction is imposed on the hardening law. A numerical method is only required to evaluate ordinary integrals. The solution is facilitated using Lagrangian coordinates. The second part of the paper is regarded as an alternative to the finite element design of tube hydroforming processes, restricted to rather simple final shapes. An advantage of this approach is that the hardening law is not required for calculating many process parameters. Therefore, the corresponding design is universally valid for all strain hardening materials if these parameters are of concern. In particular, the prediction of fracture initiation at the outer surface is independent of the hardening law for widely used ductile fracture criteria. The inner pressure is the only essential process parameter whose value is controlled by the hardening law.

## 1. Introduction

Approximate semi-analytical solutions for rigid plastic models at large strains are useful for quick analysis and design of metal forming processes. Only a few solutions are available for strain hardening materials. A solution for the radial planar flow of linear hardening material has been provided in [1]. This solution has been extended to axisymmetric flow in [2]. The solutions above have been adopted to analyze the extrusion and drawing of sheets, rods, and tubes [3,4]. The continued plane strain compression of a thin strip has been considered in [5]. A solution for the pure plane strain bending of a sheet has been derived in [6]. This solution has been extended to plane strain bending under tension in [7]. No restriction on the strain hardening law has been imposed in [6,7]. Paper [8] has further extended the solution to include kinematic hardening.

The present paper provides an exact semi-analytical solution for a hollow cylinder subject to expansion and elongation/contraction. The constitutive equations are the Mises-type yield criterion and its associated flow rule. The tensile yield stress is an arbitrary function of the equivalent strain. The solution is facilitated using Lagrangian coordinates. A numerical method is only necessary to evaluate ordinary integrals.

It has been demonstrated in [9] that a plane strain solution for an expanding two-layer hollow cylinder can be useful for analyzing tube hydroforming processes. However, the loading path is very important for designing such processes (for example, [10]). In particular, the effect of process parameters on the formability of small-diameter ZM21 magnesium alloy tubes in warm tube hydroforming has been studied in [11]. A finite element optimization of a tube hydroforming process has been presented in [12]. The effect of process parameters on stress distributions and thickness reduction has been revealed. Taguchi’s method has been employed in [13] for multi-objective optimization of critical process parameters, assuming mechanical properties of IF steel. Paper [14] compares the axial and fixed feed conditions using two failure criteria. The design for hydro-flanging processes proposed in [15] accounts for the loading path and the punch’s shape. The tube’s material is aluminum alloy A6063-T0. An in-process feedback control system has been developed in [16]. The system has been tested on aluminum alloys 5049-O and 6060-T6. All the studies above are based on finite element solutions. Simplified stress analyses of tube hydroforming processes have been presented in [17,18]. The present paper proposes an alternative to FEM-based designs using the new theoretical solution. It extends the approach [9] to include the loading path in the design. A significant portion of the solution is independent of the hardening law. In particular, this law is not required to predict fracture initiation at the outer surface if widely used ductile fracture criteria are employed.

## 2. Statement of the Problem

**Figure 1:**
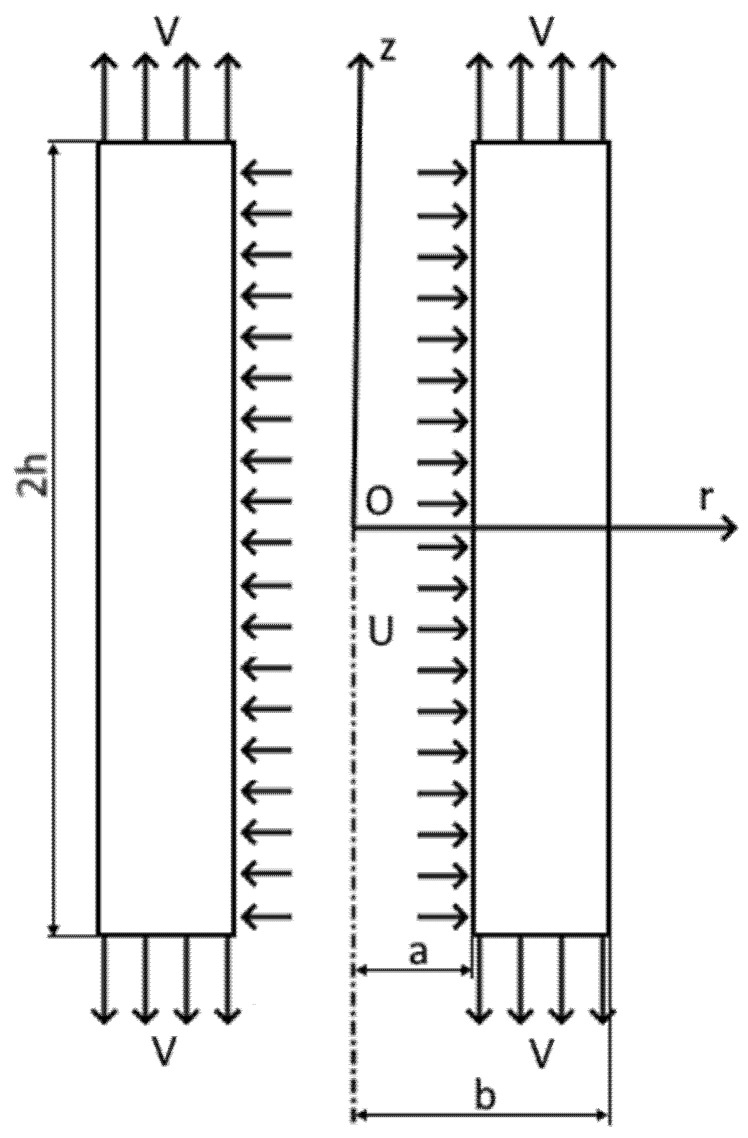
A schematic diagram of the boundary value problem.

A tube is subject to simultaneous axial elongation or contraction. The initial inner and outer radii of the tube are $a_{0}$ and $b_{0}$, respectively. The initial length of the tube is $2h_{0}$. The inner radius expands at the velocity *U*, and the tube elongates at the velocity 2*V*. Both are constant. The tube shortens if V<0. The current inner radius, the current outer radius, and the current half-length of the tubes are denoted as *a*, *b*, and *h*, respectively. It is natural to choose a cylindrical coordinate system $\left(r,\theta ,z\right)$ whose *z*-axis coincides with the tube’s axis. Moreover, the plane z=0 coincides with the process’ plane of symmetry. A current configuration of the tube is shown in Figure 1.

The solution is independent of θ. Let $u_{r}$ and $u_{z}$ be the radial and axial velocities, respectively. The circumferential velocity vanishes. Due to symmetry, it is sufficient to consider the domain 0≤z≤h. The velocity boundary conditions are

(1) $$u_{r}=U$$

for r=a,

(2) $$u_{z}=V$$

for z=h, and

(3) $$u_{z}=0$$

for z=0. 

Let $\sigma_{r}$, $\sigma_{\theta }$, and $\sigma_{z}$ be the normal stresses referred to in the cylindrical coordinate system. It is assumed that these stresses are the principal stresses. The hydrostatic stress is defined as

(4) $$\sigma =\frac{\sigma_{r}+\sigma_{\theta }+\sigma_{z}}{3}.$$

The stress boundary condition is

(5) $$\sigma_{r}=0$$

for r=b. The inner pressure is determined from the equation:

(6) $$P=-{\left.\sigma_{r}\right|}_{r=a}.$$

The tube’s material is assumed to be rigid/plastic, strain hardening. Under the conditions above, the von Mises yield criterion reads

(7) $$\sigma_{eq}=\frac{3}{2}\left(s_{r}^{2}+s_{\theta }^{2}+s_{z}^{2}\right)=\sigma_{0}^{2}\Phi^{2}\left(\varepsilon_{eq}\right).$$

Here, $s_{r}=\sigma_{r}-\sigma$, $s_{\theta }=\sigma_{\theta }-\sigma$, $s_{z}=\sigma_{z}-\sigma$, $\varepsilon_{eq}$ is the equivalent strain, $\sigma_{0}$ is the yield stress in uniaxial tension at $\varepsilon_{eq}=0$, and $\Phi \left(\varepsilon_{eq}\right)$ is an arbitrary function of its argument satisfying the conditions $\Phi \left(0\right)=1$ and $d\Phi \left(\varepsilon_{eq}\right)/d\varepsilon_{eq}\geq 0$ for all $\varepsilon_{eq}$. The following equation defines the equivalent strain:

(8) $$\frac{d\varepsilon_{eq}}{dt}=\xi_{eq}$$

where *t* is the time, d/dt denotes the convected derivative and $\xi_{eq}$ is the equivalent strain rate. In the case under consideration, the latter is defined as

(9) $$\xi_{eq}=\sqrt{\frac{2}{3}}\sqrt{\xi_{r}^{2}+\xi_{\theta }^{2}+\xi_{z}^{2}}.$$

Here, $\xi_{r}$, $\xi_{\theta }$ and $\xi_{z}$ are the radial, circumferential, and axial strain rates, respectively. These strain rates are expressed through the velocity components as

(10) $$\xi_{r}=\frac{\partial u_{r}}{\partial r},\;\xi_{\theta }=\frac{u_{r}}{r},\;\xi_{z}=\frac{\partial u_{z}}{\partial z}.$$

The plastic flow rule associated with the yield criterion in Equation (7) is

(11) $$\xi_{r}=\lambda s_{r},\;\xi_{\theta }=\lambda s_{\theta },\;\xi_{z}=\lambda s_{z}.$$

Here, λ is a non-negative multiplier. The equivalent strain vanishes at the initial instant. Therefore, the initial condition to Equation (8) is

(12) $$\varepsilon_{eq}=0$$

at $a=a_{0}$.

The only equilibrium equation that is not identically satisfied is

(13) $$\frac{\partial \sigma_{r}}{\partial r}+\frac{\sigma_{r}-\sigma_{\theta }}{r}=0.$$

## 3. Solution

The boundary conditions Equations (1) and (5) suggest that the solution, except the axial velocity, is independent of *z*. The solution starts with the velocity field. The axial velocity satisfying the conditions Equations (2) and (3) is taken as

(14) $$\frac{u_{z}}{V}=\frac{z}{h}.$$

A consequence of the plastic flow rule Equation (11) is the incompressibility equation. The latter reads $\xi_{r}+\xi_{\theta }+\xi_{z}=0$. Using Equations (10) and (14), one transforms this equation to

(15) $$\frac{\partial u_{r}}{\partial r}+\frac{u_{r}}{r}+\frac{V}{h}=0.$$

This equation can be immediately integrated to give

(16) $$u_{r}=\frac{C}{r}-\frac{r}{2}\frac{V}{h}$$

where *C* is constant. Using the boundary condition Equations (1) and (16), one can find:

(17) $$C=Ua+\frac{a^{2}}{2}\frac{V}{h}.$$

Then, Equation (16) becomes

(18) $$\frac{u_{r}}{V}=\frac{Ua}{Vr}+\frac{r}{2h}\left(\frac{a^{2}}{r^{2}}-1\right).$$

By definition,

(19) $$\frac{dr}{dt}=u_{r},\;\;\frac{dh}{dt}=V,\;\;\mathrm{and}\;\;\frac{da}{dt}=U.$$

Equations (18) and (19) combine to give

(20) $$\frac{dr}{da}=\frac{a}{r}+\frac{r}{2h}\left(\frac{a^{2}}{r^{2}}-1\right)\frac{V}{U}\;\;\mathrm{and}\;\;\frac{dh}{da}=\frac{V}{U}.$$

Integrating the second equation yields

(21) $$h=\frac{V}{U}(a-a_{0})+h_{0}.$$

This solution satisfies the initial condition $h=h_{0}$ at $a=a_{0}$. Using (21), one transforms the first equation in Equation (20) to

(22) $$\frac{dr}{da}=\frac{a}{r}+\frac{r}{2\left(a-a_{0}+h_{0}U/V\right)}\left(\frac{a^{2}}{r^{2}}-1\right).$$

It is convenient to introduce the following dimensionless quantities:

(23) $$\eta =\rho^{2}=\frac{r^{2}}{a_{0}^{2}},\;\;\alpha =\frac{a}{a_{0}},\;\;\beta =\frac{b_{0}}{a_{0}},\;\;\mathrm{and}\;\;s=\frac{h_{0}}{a_{0}}\frac{U}{V}.$$

Then, Equation (22) transforms to the following linear differential equation:

(24) $$\frac{d\eta }{d\alpha }=2\alpha +\frac{\alpha^{2}-\eta }{\alpha -1+s}.$$

Its general solution is

(25) $$\eta =\frac{\alpha^{3}+\left(s-1\right)\alpha^{2}+C_{0}}{\alpha -1+s}$$

where $C_{0}$ is constant. Let *R* be the Lagrangian coordinate such that ρ=R at the initial instant (i.e., at α=1). Then, it follows from Equations (23) and (25) that $C_{0}=s\left(R^{2}-1\right)$. Using this equation to eliminate $C_{0}$ in Equation (25), one gets

(26) $$\rho =\sqrt{\eta }=\sqrt{\frac{\alpha^{3}+\left(s-1\right)\alpha^{2}+s\left(R^{2}-1\right)}{\alpha -1+s}}.$$

The strain rate components in the cylindrical coordinate system are determined from Equations (10), (14), (18) and (23) as

(27) $$\xi_{z}=\frac{U}{a_{0}\left(\alpha -1+s\right)},\;\xi_{r}=-\frac{U}{a_{0}}\left[\frac{\alpha }{\eta }+\frac{1}{2\left(\alpha -1+s\right)}\left(\frac{\alpha^{2}}{\eta }+1\right)\right],\;\xi_{\theta }=\frac{U}{a_{0}}\left[\frac{\alpha }{\eta }+\frac{1}{2\left(\alpha -1+s\right)}\left(\frac{\alpha^{2}}{\eta }-1\right)\right].$$

Substituting Equation (27) into Equation (9) yields the equivalent strain rate as

(28) $$\xi_{eq}=\frac{U}{a_{0}\left|\alpha -1+s\right|}\sqrt{1+\frac{\alpha^{2}}{3\eta^{2}}{\left(3\alpha -2+2s\right)}^{2}}.$$

Using Equation (26), one can rewrite Equation (28) in the Lagrangian coordinates as

(29) $$\xi_{eq}=\frac{U}{a_{0}\left|\alpha -1+s\right|}\sqrt{1+\frac{\alpha^{2}}{3}\frac{{\left(3\alpha -2+2s\right)}^{2}{\left(\alpha -1+s\right)}^{2}}{{\left[\alpha^{3}+\left(s-1\right)\alpha^{2}+s\left(R^{2}-1\right)\right]}^{2}}}.$$

Since $d\varepsilon_{eq}/dt=\partial \varepsilon_{eq}/\partial t$ in the Lagrangian coordinates, it follows from Equations (8), (19), (23) and (29) that

(30) $$\frac{\partial \varepsilon_{eq}}{\partial \alpha }=\frac{1}{\left|\alpha -1+s\right|}\sqrt{1+\frac{\alpha^{2}}{3}\frac{{\left(3\alpha -2+2s\right)}^{2}{\left(\alpha -1+s\right)}^{2}}{{\left[\alpha^{3}+\left(s-1\right)\alpha^{2}+s\left(R^{2}-1\right)\right]}^{2}}}.$$

Using Equation (23), one can represent the solution of this equation satisfying the initial condition Equation (12) as

(31) $$\varepsilon_{eq}=\int_{1}^{\alpha }\frac{1}{\left|\gamma -1+s\right|}\sqrt{1+\frac{\gamma^{2}}{3}\frac{{\left(3\gamma -2+2s\right)}^{2}{\left(\gamma -1+s\right)}^{2}}{{\left[\gamma^{3}+\left(s-1\right)\gamma^{2}+s\left(R^{2}-1\right)\right]}^{2}}}d\gamma .$$

The yield criterion Equation (7) is satisfied by the following substitution:

(32) $$\begin{array}{l} s_{r}=-\frac{2}{3}\sigma_{0}\Phi \left(\varepsilon_{eq}\right)\sin\psi ,\\ s_{\theta }=\frac{1}{3}\sigma_{0}\Phi \left(\varepsilon_{eq}\right)\left(\sin\psi +\sqrt{3}\cos\psi \right),\\ s_{z}=\frac{1}{3}\sigma_{0}\Phi \left(\varepsilon_{eq}\right)\left(\sin\psi -\sqrt{3}\cos\psi \right). \end{array}$$

Here, ψ is a new unknown function of R and α. It follows from the associated flow rule Equation (11) that

(33) $$\frac{\xi_{r}}{s_{r}}=\frac{\xi_{\theta }}{s_{\theta }}=\frac{\xi_{z}}{s_{z}}.$$

Equations (32) and (33) combine to give

(34) $$\frac{\xi_{\theta }}{\xi_{r}}=-\frac{1}{2}\left(1+\sqrt{3}\cot\psi \right),\;\frac{\xi_{\theta }}{\xi_{z}}=\frac{\tan\psi +\sqrt{3}}{\tan\psi -\sqrt{3}}.$$

Substituting Equation (27) into each of the equations in Equation (34) and eliminating η by means of Equation (26) leads to

(35) $$\cot\psi =\frac{2\alpha^{2}\left(s-1\right)+2\alpha {\left(s-1\right)}^{2}+3s\left(\mu -1\right)}{\sqrt{3}\left[4\alpha^{3}+6\alpha^{2}\left(s-1\right)+2\alpha {\left(s-1\right)}^{2}+s\left(\mu -1\right)\right]}$$

where $\mu =R^{2}$. Equation (26) can be solved for μ as

(36) $$\mu =1+\frac{\rho^{2}\left(\alpha -1+s\right)-\alpha^{3}-\left(s-1\right)\alpha^{2}}{s}.$$

Then,

(37) $$\frac{\partial \mu }{\partial \rho }=\frac{2\left(\alpha -1+s\right)\rho }{s}.$$

Since $\sigma_{r}-\sigma_{\theta }=s_{r}-s_{\theta }$, upon substitution from Equations (23) and (32), Equation (13) becomes

(38) $$\frac{\partial \sigma_{r}}{\partial \rho }-\frac{\sigma_{0}\left(\sqrt{3}\sin\psi +\cos\psi \right)\Phi \left(\varepsilon_{eq}\right)}{\sqrt{3}\rho }=0.$$

Replacing here differentiation with respect to ρ with differentiation with respect to μ utilizing Equation (37) and eliminating ρ employing Equation (26), one gets

(39) $$\frac{\partial \sigma_{r}}{\partial \mu }=\frac{\sigma_{0}s\left(\cos\psi +\sqrt{3}\sin\psi \right)\Phi \left(\varepsilon_{eq}\right)}{2\sqrt{3}\left[\alpha^{3}+\left(s-1\right)\alpha^{2}+s\left(\mu -1\right)\right]}.$$

The right-hand side of this equation is a function of μ and α due to Equations (31) and (35). In the Lagrangian coordinates, the boundary condition Equation (5) becomes $\sigma_{r}=0$ for R=β (or $\mu =\beta^{2}$). The solution of Equation (39) satisfying this boundary condition is

(40) $$\frac{\sigma_{r}}{\sigma_{0}}=\frac{s}{2\sqrt{3}}\int_{\beta^{2}}^{\mu }\frac{\Phi \left(\varepsilon_{eq}\right)\left(\sqrt{3}\sin\psi +\cos\psi \right)}{\left[\alpha^{3}+\left(s-1\right)\alpha^{2}+s\left(\chi -1\right)\right]}d\chi .$$

The dimensionless inner pressure is determined from this solution and Equation (6) as

(41) $$p=\frac{P}{\sigma_{0}}=\frac{s}{2\sqrt{3}}\int_{1}^{\beta^{2}}\frac{\Phi \left(\varepsilon_{eq}\right)\left(\sqrt{3}\sin\psi +\cos\psi \right)}{\left[\alpha^{3}+\left(s-1\right)\alpha^{2}+s\left(\mu -1\right)\right]}d\mu .$$

The integrals in Equations (31) and (40) can be evaluated numerically. Then, the other stress components are determined from Equations (32) and (35) as

(42) $$\begin{array}{l} \sigma =\sigma_{r}-s_{r}=\sigma_{r}+\frac{2}{3}\sigma_{0}\Phi \left(\varepsilon_{eq}\right)\sin\psi ,\\ \sigma_{\theta }=s_{\theta }+\sigma =\sigma_{r}+\sigma_{0}\Phi \left(\varepsilon_{eq}\right)\left(\sin\psi +\frac{\cos\psi }{\sqrt{3}}\right),\\ \sigma_{z}=s_{z}+\sigma =\sigma_{r}+\sigma_{0}\Phi \left(\varepsilon_{eq}\right)\left(\sin\psi -\frac{\cos\psi }{\sqrt{3}}\right). \end{array}$$

Figure 1 shows the velocity *V*. It is assumed that it is the velocity of the punch. In this case, the axial punch force is given by

(43) $$Q=2\pi \int_{a}^{b}\sigma_{z}rdr.$$

It follows from Equations (23) and (36) that

(44) $$rdr=\frac{a_{0}^{2}s}{2\left(\alpha -1+s\right)}d\mu .$$

Using Equations (42) and (44), one can determine the dimensionless axial punch force from Equation (43) as

(45) $$q=\frac{Q}{\pi \sigma_{0}a_{0}^{2}}=\frac{s}{\left(\alpha -1+s\right)}\int_{1}^{\beta^{2}}\left[\frac{\sigma_{r}}{\sigma_{0}}+\Phi \left(\varepsilon_{eq}\right)\left(\sin\psi -\frac{\cos\psi }{\sqrt{3}}\right)\right]d\mu .$$

Upon substitution from Equations (31), (35) and (40), the integrand becomes a known function of μ. The integral should be evaluated numerically.

The plane strain solution is a particular case of the solution above. However, in this case, V=0, and, as follows from Equation (23), *s* approaches infinity or negative infinity. The easiest way to get the plane strain solution is to replace *s* with 1/p and put p=0. This solution has been derived in [9]. 

## 4. Application to Tube Hydroforming

The geometric model shown in Figure 1 has been successfully applied to some tube hydroforming processes assuming that V=0 [9]. However, the loading path is important for tube hydroforming design ([10] and the list of references therein). The present solution allows for the loading path to be considered through s-values. The present section contains numerical solutions. All these solutions have been found at β=1.08. This value is typical for tube hydroforming processes [19].

### 4.1. Inner Pressure

**Figure 2:**
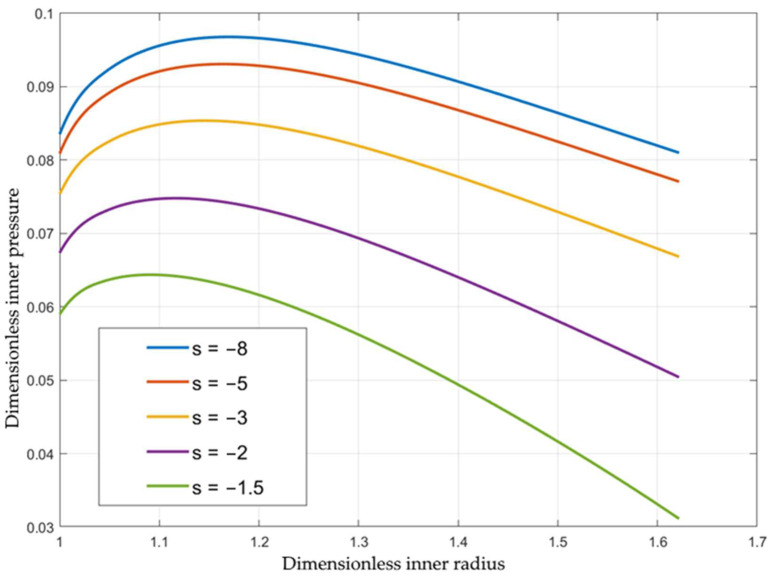
Variation of the inner pressure with the inner radius for several loading paths.

The inner pressure is one of the most important process parameters. Its magnitude depends on the loading path, geometric parameters, and material properties. Using the solution above, one needs to evaluate the integral in Equation (41) for calculating the dimensionless pressure. Figure 2 shows the effect of the loading path on the variation of the inner pressure with the inner radius for St37 steel, assuming that $\Phi \left(\varepsilon_{eq}\right)=1+2.4\varepsilon_{eq}^{0.812}$ [19]. As expected, the pressure increases as $\left|V\right|$ decreases (i.e., $\left|s\right|$ increases). It is important to get the dependence of the inner pressure on the axial feeding. The latter is defined as $h_{0}-h.$

Using Equations (21) and (23), one may define the dimensionless axial feeding as

(46) $$\omega =\frac{h_{0}-h}{h_{0}}=\frac{1-\alpha }{s}.$$

**Figure 3:**
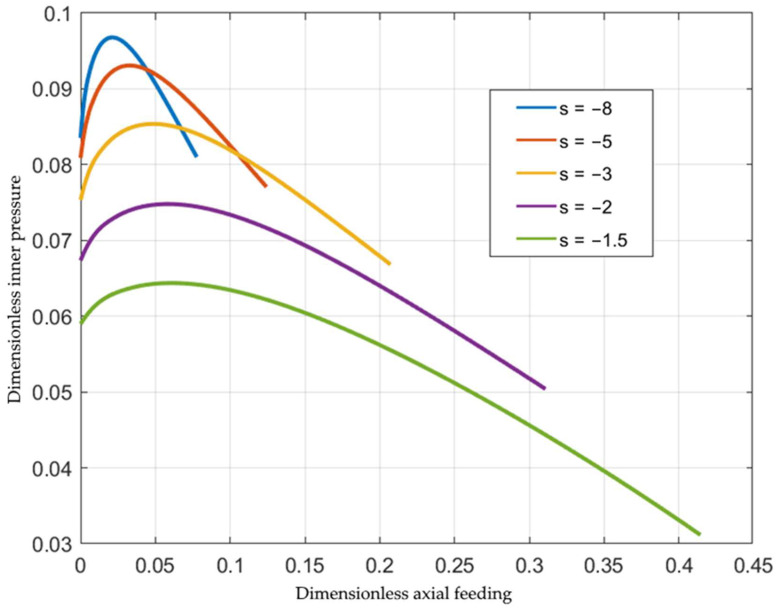
Variation of the inner pressure with the axial feeding for several loading paths.

**Figure 4:**
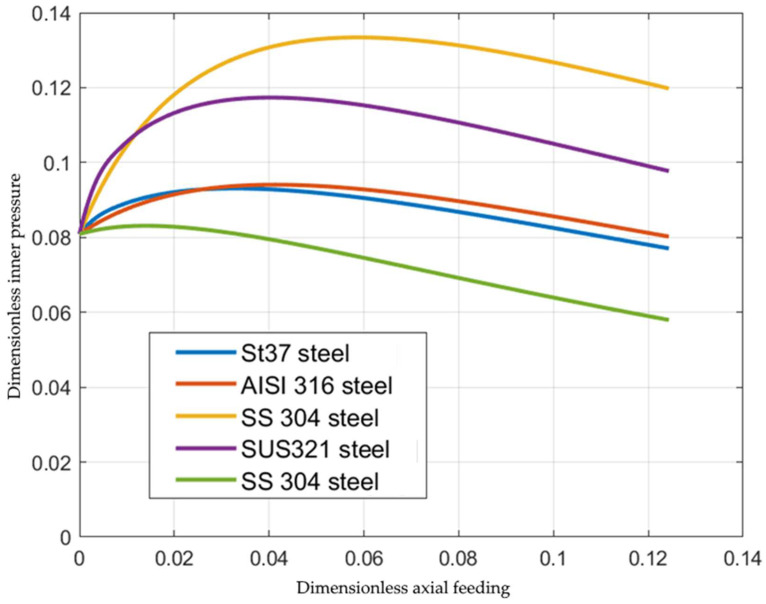
Variation of the inner pressure with the axial feeding for several materials (St37 steel [19], AISI 316 steel [20], SS 304 steel [21], SUS321 steel [22], SS 304 steel [23]).

The curves in Figure 2 are re-plotted in Figure 3 utilizing Equation (46). The function $\Phi \left(\varepsilon_{eq}\right)$ involved in Equation (7) greatly affects the inner pressure. Figure 4 compares the inner pressure at s=−5 for five materials: St37 steel, AISI 316L steel ($\Phi \left(\varepsilon_{eq}\right)={\left(1+6.29\varepsilon_{eq}\right)}^{0.634}$) [20], SS-304 steel ($\Phi \left(\varepsilon_{eq}\right)={\left(1+16.67\varepsilon_{eq}\right)}^{0.584}$) [21], SUS321 steel ($\Phi \left(\varepsilon_{eq}\right)=1+3.15\varepsilon_{eq}^{0.66}$) [22], and SS-304 steel ($\Phi \left(\varepsilon_{eq}\right)={\left(1+7.62\varepsilon_{eq}\right)}^{0.361}$) [23]. Surprisingly, the most significant difference occurs in the case of two functions for SS-304 provided in two different sources. The reason for this difference in the present solution is that the stress–strain curves are very different.

Figure 3 illustrates a one-parameter family of loading paths in the inner pressure-axial feeding space, with *s* being the parameter. Similar families can be found in other spaces using the solution in Section 3. In particular, the space of the principal strains is often used to describe metal forming processes. In the case under consideration, the principal strains coincide with the strains in the cylindrical coordinate system. Using Equation (26), one can integrate the equations in Equation (27) to get

$$\begin{array}{l} \varepsilon_{z}=\ln\left(\frac{s-1+\alpha }{s}\right),\;\varepsilon_{\theta }=\frac{1}{2}\ln\left[\frac{\alpha^{3}+\left(s-1\right)\alpha^{2}+s\left(R^{2}-1\right)}{R^{2}\left(\alpha -1+s\right)}\right],\\ \varepsilon_{r}=\ln\left(\frac{s}{s-1+\alpha }\right)-\frac{1}{2}\ln\left[\frac{\alpha^{3}+\left(s-1\right)\alpha^{2}+s\left(R^{2}-1\right)}{R^{2}\left(\alpha -1+s\right)}\right]. \end{array}$$

For each *s*, the principal strain path can be determined from these equations eliminating *α*. The path depends on the Lagrangian coordinate. 

The solution in Section 3 is semi-analytical. It does not require experimental verification. However, its applicability to modeling tube hydroforming processes does. Experimental data should include the value of *s*, which is usually unavailable. The experimental verification below is based on data presented in [24] for aluminum alloy A1070. The essential geometric parameters are $a_{0}=19$ mm and $b_{0}=20$ mm. The hardening law for this material has been provided in [25] using the Holloman equation. This equation is incompatible with the present solution because it results in $\sigma_{0}=0$. However, it has been shown in [9] that the Holloman equation can be replaced with the Ludwik equation. In particular, $\sigma_{0}=32.2$ MPa and $\Phi \left(\varepsilon_{eq}\right)=1+3.6\varepsilon_{eq}^{0.37}$. Paper [24] provides the variation of the inner pressure and the axial force with $\Delta b=b-b_{0}$. This quantity is determined from Equation (26) as

$$\frac{\Delta b}{a_{0}}=\sqrt{\frac{\alpha^{3}+\left(s-1\right)\alpha^{2}+s\left(\beta^{2}-1\right)}{\alpha -1+s}}-\beta .$$

**Figure 5:**
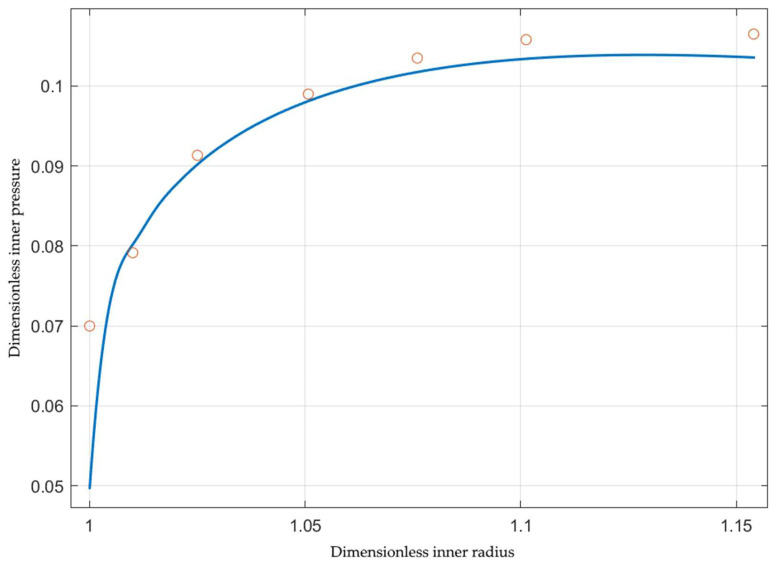
Comparison of the theoretical prediction of the inner pressure and experiment [24]. The solid line corresponds to the theoretical prediction and the circles to the experiment.

**Figure 6:**
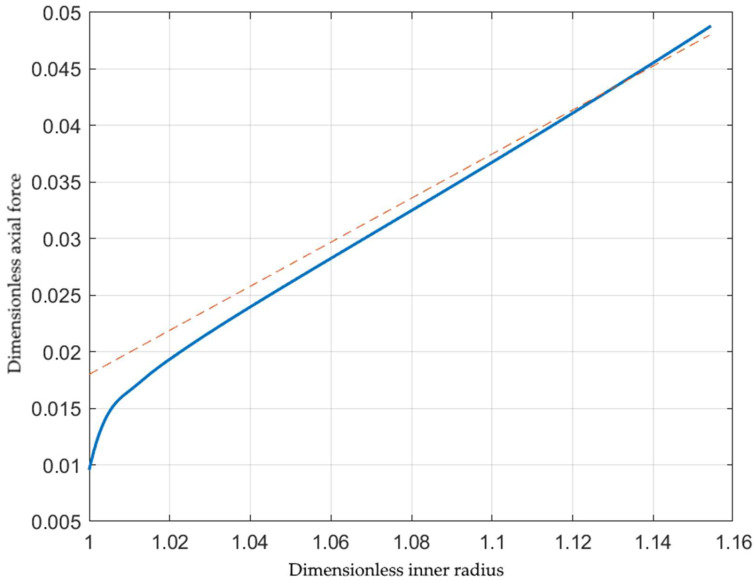
Comparison of the theoretical prediction of the axial force and experiment [24]. The solid line corresponds to the theoretical prediction and the broken line to the experiment.

The theoretical dimensionless inner radius and axial force depend on *s*. The latter has been calculated to approximate both experimental curves in [24] closely. It has been found that s=−3.02. Figure 5 compares the experimental and theoretical dimensionless pressures. The latter has been found using Equation (41). The experimental values are shown as a discrete function. A small range of the experimental curve corresponding to elastic loading has been disregarded. A practically straight line represents the axial force in [24] if this range is disregarded. The line corresponding to the dimensionless axial force is shown by the broken line in Figure 6. The theoretical dimensionless axial force has been found using Equation (45). The solid line represents this force in Figure 6. It is seen from Figure 5 and Figure 6 that the theoretical prediction is rather accurate, taking into account the simplicity of the solution.

### 4.2. Wall Thickness

The wall thickness is one of the most important geometric parameters. Its initial value is $H_{0}=b_{0}-a_{0}$. The Lagrangian coordinates of the inner and outer radii of the tube are R=1 and R=β, respectively. Then, the current thickness of the wall is determined from Equation (26) as

(47) $$H=H_{0}{\left(\beta -1\right)}^{-1}\left[\sqrt{\frac{\alpha^{3}+\left(s-1\right)\alpha^{2}+s\left(\beta^{2}-1\right)}{\alpha -1+s}}-\sqrt{\frac{\alpha^{3}+\left(s-1\right)\alpha^{2}}{\alpha -1+s}}\right].$$

Equations (46) and (47) supply the dependence of $H/H_{0}$ on the axial feeding in parametric form with α being the parameter. 

The wall thinning defined as $\delta =1-H/H_{0}$ is sometimes adopted as the failure limit in tube hydroforming analysis and design [21]. Let $\delta_{*}$ be the critical value of δ. It follows from Equation (47) that

(48) $$\delta_{*}=1-{\left(\beta -1\right)}^{-1}\left[\sqrt{\frac{\alpha_{*}^{3}+\left(s-1\right)\alpha_{*}^{2}+s\left(\beta^{2}-1\right)}{\alpha_{*}-1+s}}-\sqrt{\frac{\alpha_{*}^{3}+\left(s-1\right)\alpha_{*}^{2}}{\alpha_{*}-1+s}}\right].$$

**Figure 7:**
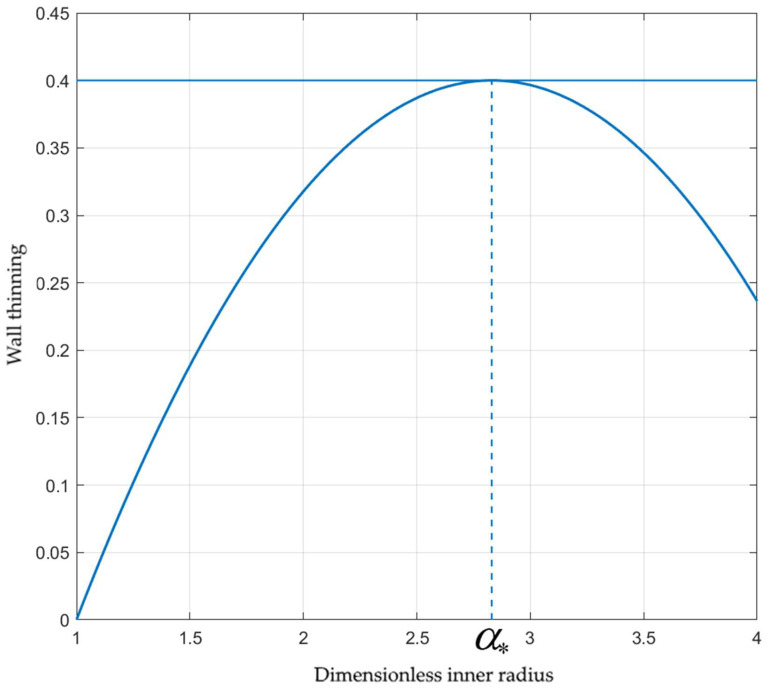
Geometric illustration of the special solution to Equation (48).

**Figure 8:**
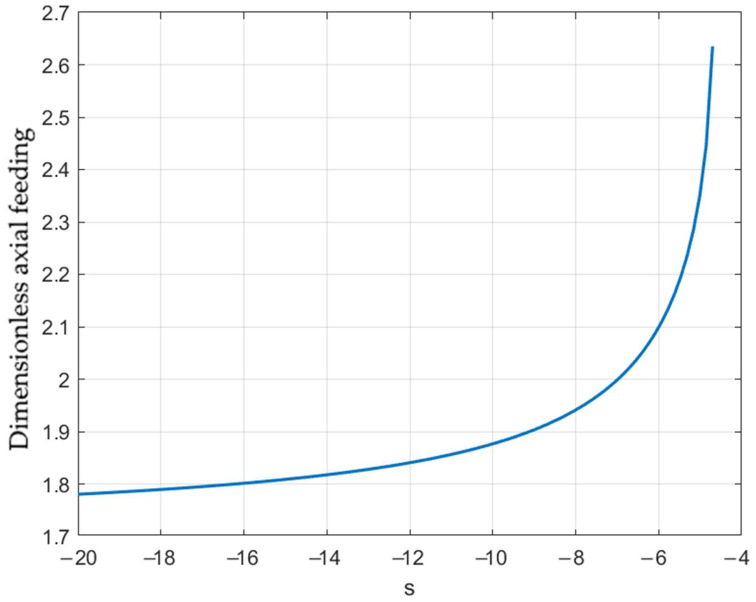
Effect of the loading path on fracture initiation.

Here, $\alpha_{*}$ is the critical value of α corresponding to $\delta =\delta_{*}$. At a given value of $\delta_{*}$, this equation should be solved for $\alpha_{*}$. The right-hand side of Equation (48), considered to be the function of $\alpha_{*}$, may attain a local maximum. If this maximum is less than $\delta_{*}$, the equation has no solution. In this case, the mode of failure above does not occur. Therefore, it is important to find such a special solution to Equation (48) that its right-hand side attains the local maximum at the same value of $\alpha_{*}$. To satisfy these two conditions, one should allow for *s* or β to be varied. In what follows, β is kept constant. Figure 7 illustrates the special solution if $\delta_{*}=0.4$, which is a typical value [21]. The corresponding values of the other quantities are $s=s_{*}\approx -4.66$ and $\alpha_{*}\approx 2.83$. Equation (48) has no solution if $s>s_{*}$. Using Equation (46), one can rewrite the right-hand side of Equation (48) in terms of the axial feeding. The dependence of ω at which $\delta =\delta_{*}=0.4$ on s is depicted in Figure 8.

### 4.3. Damage

Damage mechanics models are often used to predict fracture initiation in tube hydroforming processes [19,21]. A wide class of damage mechanics models is based on ductile fracture criteria [26]. The present paper considers two ductile fracture criteria. One of these criteria has been proposed in [27] and modified in [28]. The modified criterion reads

(49) $$\int_{0}^{\varepsilon_{eq}^{f}}\frac{\sigma_{m}}{\sigma_{eq}}d\varepsilon_{eq}=C_{1}.$$

Here, $\sigma_{m}$ is the largest principal stress, $\varepsilon_{eq}^{f}$ is the equivalent strain to fracture, and $C_{1}$ is a constitutive parameter. The other criterion reads [29]

(50) $$\int_{0}^{\varepsilon_{eq}^{f}}\left(1+C_{2}\frac{\sigma }{\sigma_{eq}}\right)d\varepsilon_{eq}=C_{3}.$$

Here, $C_{2}$ and $C_{3}$ are constitutive parameters. The integrals in Equations (49) and (50) are taken over the strain path. 

Fracture often initiates at the outer surface [19]. It follows from Equation (5) that

(51) $$\sigma +s_{r}=0$$

at the fracture initiation site. The largest principal stress is the circumferential stress. Using Equations (32) and (51), one finds

(52) $$\sigma_{m}=\sigma_{\theta }=s_{\theta }+\sigma =s_{\theta }-s_{r}=\sigma_{0}\Phi \left(\varepsilon_{eq}\right)\left(\sin\psi +\frac{\cos\psi }{\sqrt{3}}\right).$$

Substituting Equations (7) and (52) into Equation (49) gives

(53) $$\int_{0}^{\varepsilon_{eq}^{f}}\left(\sin\psi +\frac{\cos\psi }{\sqrt{3}}\right)d\varepsilon_{eq}=C_{1}.$$

Here, ψ is understood to be calculated from Equation (35) at $\mu =\beta^{2}$. Using Equation (30), one can rewrite Equation (53) as

(54) $$\int_{1}^{\alpha_{f}}\left(\sin\psi +\frac{\cos\psi }{\sqrt{3}}\right)\frac{d\varepsilon_{eq}}{d\alpha }d\alpha =\frac{1}{\sqrt{3}}\int_{1}^{\alpha_{f}}\frac{\left(\sqrt{3}\sin\psi +\cos\psi \right)}{\left|\alpha -1+s\right|}\sqrt{1+\frac{\alpha^{2}}{3}\frac{{\left(3\alpha -2+2s\right)}^{2}{\left(\alpha -1+s\right)}^{2}}{{\left[\alpha^{3}+\left(s-1\right)\alpha^{2}+s\left(\beta^{2}-1\right)\right]}^{2}}}d\alpha =C_{1}.$$

**Figure 9:**
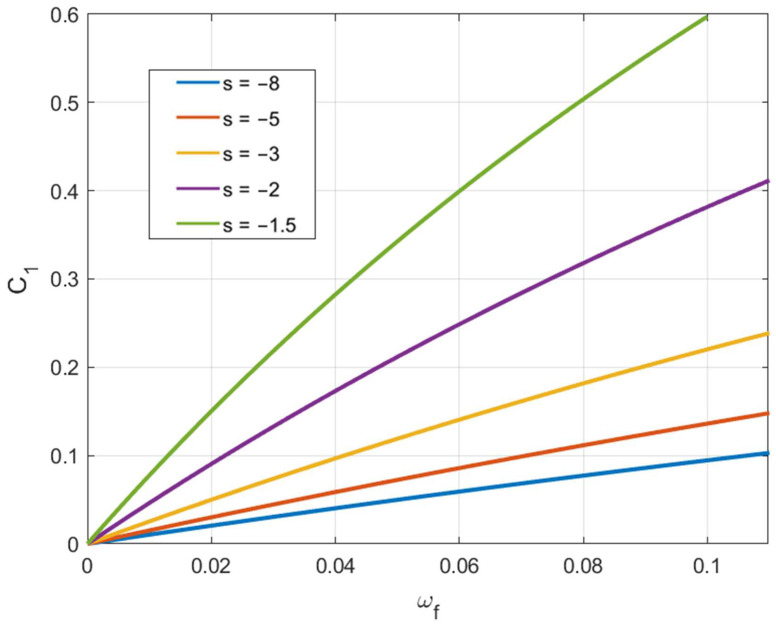
Variation of $C_{1}$ involved in Equation (54) with the axial feeding for several loading paths.

Here, $\alpha_{f}$ is the value of α at fracture initiation. The corresponding value of ω can be found from Equation (46) and is denoted as $\omega_{f}$. Figure 9 depicts $C_{1}$ calculated from (54) for several loading paths. These curves allow one to find the axial feeding at which fracture occurs for a given value of $C_{1}$.

Substituting Equations (7), (32) and (51) into Equation (50) gives

(55) $$\int_{0}^{\varepsilon_{eq}^{f}}\left(1-C_{2}\frac{s_{r}}{\sigma_{eq}}\right)d\varepsilon_{eq}=\int_{0}^{\varepsilon_{eq}^{f}}\left(1+\frac{2C_{2}}{3}\sin\psi \right)d\varepsilon_{eq}=C_{3}.$$

As before, using Equation (30), one can rewrite Equation (55) as

(56) $$\frac{1}{3}\int_{1}^{\alpha_{f}}\frac{\left(3+2C_{2}\sin\psi \right)}{\left|\alpha -1+s\right|}\sqrt{1+\frac{\alpha^{2}}{3}\frac{{\left(3\alpha -2+2s\right)}^{2}{\left(\alpha -1+s\right)}^{2}}{{\left[\alpha^{3}+\left(s-1\right)\alpha^{2}+s\left(\beta^{2}-1\right)\right]}^{2}}}d\alpha =C_{3}.$$

It is convenient to represent this equation as

(57) $$\Lambda_{1}\left(\alpha_{f}\right)+\Lambda_{2}\left(\alpha_{f}\right)C_{2}=C_{3}$$

where

(58) $$\begin{array}{l} \Lambda_{1}\left(\alpha_{f}\right)=\int_{1}^{\alpha_{f}}\frac{1}{\left|\alpha -1+s\right|}\sqrt{1+\frac{\alpha^{2}}{3}\frac{{\left(3\alpha -2+2s\right)}^{2}{\left(\alpha -1+s\right)}^{2}}{{\left[\alpha^{3}+\left(s-1\right)\alpha^{2}+s\left(\beta^{2}-1\right)\right]}^{2}}}d\alpha ,\\ \Lambda_{2}\left(\alpha_{f}\right)=\frac{2}{3}\int_{1}^{\alpha_{f}}\frac{\sin\psi }{\left|\alpha -1+s\right|}\sqrt{1+\frac{\alpha^{2}}{3}\frac{{\left(3\alpha -2+2s\right)}^{2}{\left(\alpha -1+s\right)}^{2}}{{\left[\alpha^{3}+\left(s-1\right)\alpha^{2}+s\left(\beta^{2}-1\right)\right]}^{2}}}d\alpha . \end{array}$$

**Figure 10:**
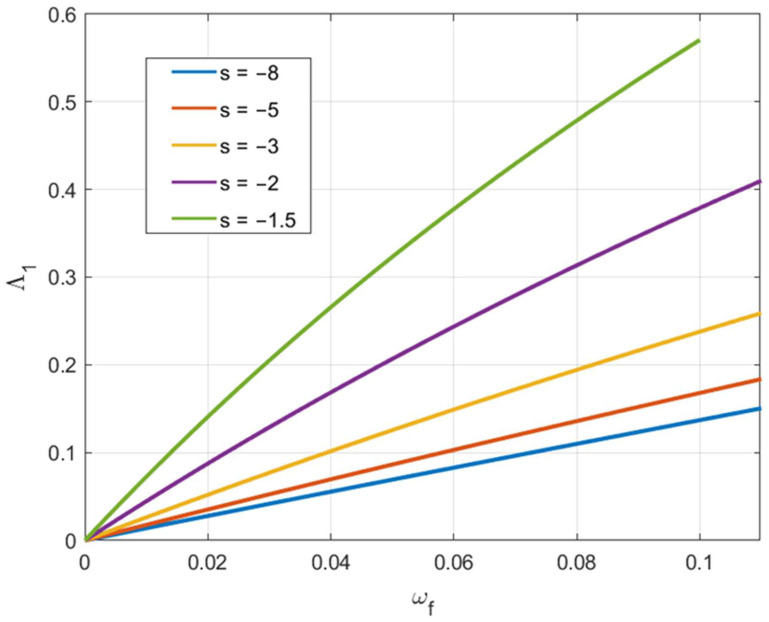
Variation of $\Lambda_{1}$ involved in Equation (57) with the axial feeding for several loading paths.

**Figure 11:**
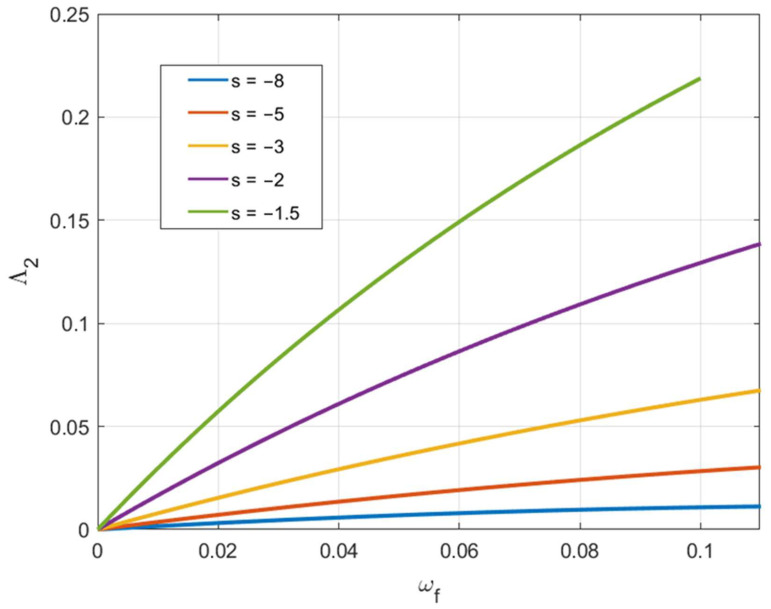
Variation of $\Lambda_{2}$ involved in Equation (57) with the axial feeding for several loading paths.

Due to Equation (46), it is possible to regard $\Lambda_{1}\left(\alpha_{f}\right)$ and $\Lambda_{2}\left(\alpha_{f}\right)$ as functions of $\omega_{f}$. Figure 10 and Figure 11 depict these functions for several loading paths. These curves and Equation (57) allow one to find the axial feeding at which fracture occurs for given values of $C_{2}$ and $C_{3}$.

## 5. Conclusions

A new rigid/plastic solution for the general isotropic hardening law has been found. The solution describes a tube’s simultaneous expansion and elongation/contraction at finite strains, giving the radial distribution of stress and strain rate at any deformation stage. It is facilitated using Lagrangian coordinates. A numerical treatment is only necessary for evaluating ordinary integrals.

The solution has been adopted for describing tube hydroforming processes. It can be used for the preliminary design of such processes. In particular, several failure criteria have been considered. The application of these criteria does not require the stress solution, which makes the final result even simpler than the general solution. The stress solution is only necessary to relate the inner pressure to other process parameters.

The solution in Section 3 is semi-analytical. It does not require experimental verification. However, its applicability to modeling tube hydroforming processes developed in Section 4 does. Experimental data should include the value of *s* introduced in Equation (23). It is usually unavailable in papers devoted to experiments. The comparison, shown in Figure 5 and Figure 6, has been made using the experimental data [24]. This paper provides the inner pressure and axial force variation as the deformation proceeds. The theoretical counterparts depend on the single parameter *s*, provided that the initial geometry and hardening law are given. Therefore, the existence of an *s*-value at which the experimental and theoretical data for both the inner pressure and the axial force coincide confirms that the semi-analytic solution can be used for the preliminary design of tube hydroforming processes. Figure 5 and Figure 6 show that the condition above is satisfied rather accurately at s=−3.02.

The theoretical solution found is useful for other applications. An experimental procedure for the inelastic response and failure materials has been developed in [30]. In particular, the solution can be combined with this procedure during the homogenous deformation stage. Another area of application is the method for repairing screen pipes using a tube hydroforming process proposed in [31].

## Data Availability

Not applicable.

## Nomenclature

*a*	inner radius
*b*	outer radius
*h*	half-length
*p*	dimensionless inner pressure
*q*	dimensionless axial punch force
(*r*, *θ*, *z*)	cylindrical coordinates
*s*	dimensionless parameter introduced in Equation (23)
*s_r_*, *s_θ_*, *s_z_*	deviatoric stress components
*t*	time
*u_r_*	radial velocity
*u_z_*	axial velocity
*H*	wall thickness
*P*	inner pressure
*R*	Lagrangian coordinate
*Q*	axial punch force
*U*	speed of expansion
*V*	speed of elongation
*α*	dimensionless inner radius
*δ*	wall thinning
*ε_eq_*	equivalent strain
*ε_r_*, *ε_θ_*, *ε_z_*	strain components
*η*	function of the polar radius introduced in Equation (23)
*ξ_eq_*	equivalent strain rate
*ξ_r_*, *ξ_θ_*, *ξ_z_*	strain rate components
*ρ*	dimensionless radius
*σ*	hydrostatic stress
*σ_eq_*	equivalent stress
*σ_r_*, *σ_θ_*, *σ_z_*	stress components
*ω*	dimensionless axial feeding
